# Notes and Notices

**Published:** 1887-02

**Authors:** 


### NOTES AND NOTICES.

SPECIAL NOTICE.—On November 5th, 1886, some one of our subscribers called at this office in the absence of the editors and paid his subscription. As he neglected to leave a memorandum of his name and address, we have not the slightest idea who it is, and are therefore unable to give the proper credit.

Will the gentleman please make good his omission? W. M. J.

CONTROLLING CONTAGIOUS DISEASES WITH COTTON.—By the use of cotton filters and respirators Dr. David Prince, of Jacksonville, Ill., proposes to keep the infectious germs from spreading out of an infected room and from entering the system of persons exposed. Cotton filters arrest the passage of all particulate matter.—*Medical Record.*

Dr. F. E. Watts has removed from Olean, N. Y., to Port Allegheny, Pa.

